# An outbreak of *Pseudomonas aeruginosa* urinary tract infections following cystoscopy traceable to a malfunctioning drying cabinet

**DOI:** 10.1016/j.infpip.2024.100378

**Published:** 2024-06-10

**Authors:** Leonie A.J. Derickx, Diana Willemse-Erix, Anne van Piggelen, Paul Steegh, A. Caroline Heijckmann, Mirjam H.A. Hermans, Thijn F. de Vocht, Peter C. Wever

**Affiliations:** a Department of Medical Microbiology, UMC Utrecht, Utrecht, The Netherlands; b Department of Medical Microbiology and Infection Control, Jeroen Bosch Hospital, ’s-Hertogenbosch, The Netherlands; c Department of Internal Medicine, Bernhoven, Uden, The Netherlands; d Department of Urology, Bernhoven, Uden, The Netherlands

**Keywords:** Urinary tract infection, *Pseudomonas aeruginosa*, Cystoscopes, Drying cabinet, Amplified fragment length polymorphism

## Abstract

**Background:**

*Pseudomonas aeruginosa* is an important bacterial pathogen, particularly as a cause of nosocomial infections in hospitalized patients. Only few reports exist in which cystoscopes were implicated as an outbreak source. We describe an investigation into the cause of a sudden increase in the number of urinary tract infections (UTI) with *P*. *aeruginosa* in patients after cystoscopy. In addition, we share the lessons learned and measures taken to reduce the risk of similar infections in the future.

**Presentation of Case:**

Over a period of two weeks the urology outpatient department noticed a UTI in four patients following cystoscopy. An investigation was started for a common source of the outbreak in the urological treatment room. Additional screening of patients revealed a total of eleven males with *P. aeruginosa* UTI following cystoscopy. The infections were found to be due to a defective drying cabinet, which lacked an alarm signaling in case of loss of airflow. Amplified fragment length polymorphism (AFLP) analysis revealed that *P. aeruginosa* isolates from three patients and six isolates from environmental cultures (including cystoscopes from the drying cabinet) genotypically belonged to one strain.

**Discussion:**

The AFLP results suggest that contaminated cystoscopes caused *P. aeruginosa* UTI in 11 patients, with the drying cabinet as site of transfer of the infective strain. To our knowledge, this is the first report describing a malfunctioning drying cabinet as source of an outbreak following cystoscopy.

**Conclusion:**

In case of concomitant *P. aeruginosa* infections, cystoscopes and drying cabinets should be suspected as a potential source. Molecular techniques are helpful in investigating the epidemiology of an outbreak.

## Introduction

*Pseudomonas aeruginosa* is an aerobic Gram-negative rod that can be isolated from soil, water, plants, humans, and animals. In humans, it is uncommonly encountered as part of the normal transient flora. Human colonization occurs mostly at moist sites such as perineum, axilla, and ear [1]. *P. aeruginosa* can cause a broad spectrum of infections involving the respiratory, gastrointestinal, and urinary tracts as well as wound infections, sepsis, and other infections. It contributes to high morbidity and mortality among patients in intensive care units, oncology departments, burn units, and surgery wards [2]. Tap water, medical equipment, hospital water systems, inadequate disinfection procedures, hospital personnel, and other patients are possible sources of *P. aeruginosa* infection in hospitals [3].

*P. aeruginosa* is responsible for 11–23% of nosocomial infections worldwide [4]. Several outbreaks of *P. aeruginosa* caused by contaminated endoscopes have been reported [[5], [6], [7]]. The incidence of urinary tract infections (UTI) after flexible cystoscopy ranges from 2 to 21.2% [8]. UTIs are common symptomatic infections occurring when bacteria enter the urethra and infect the urinary tract (mostly a bladder infection). Potential exogenous sources of organisms that can cause urologic surgery-associated infections include contaminated irrigating or disinfectant fluid, contaminated instruments and contamination inside the operating room [9]. If a cluster of cases related to the urology ward is detected, a common source associated with urologic instrumentation should be suspected [3]. Disinfecting scopes contaminated with *P. aeruginosa* can be challenging because of the ability of the bacteria to produce an exopolysaccharide biofilm that protects it from surfactant containing detergents [10].

Molecular typing is an important tool to follow transmission routes of microbial pathogens that can be used in clinical settings to discriminate ongoing epidemics of an infectious agent from incidentally increased rates [11]. Molecular techniques for analysis of clinical isolates can be helpful in investigation of the epidemiology of outbreak strains and in confirming their clonality [3].

This paper describes an investigation performed after a raised number of UTI in the urology outpatient department of Bernhoven hospital in the Netherlands, and describes the cause of the outbreak and the lessons we have learned.

## Methods

### Setting

Bernhoven is a regional general hospital in the Netherlands. The urology department has two urological treatment rooms, numbered 1 and 2. The urological scopes are cleaned by a specialized team which performs a manual pre-cleaning followed by mechanical disinfection and cleaning at the reprocessing unit for reusable medical devices. If, after disinfection, the wet scopes are not reused within four hours, they are placed in a drying cabinet. There are three drying cabinets where the scopes are hung, one in treatment room 1, one in treatment room 2 and the other at the reprocessing unit. The drying cabinets are maintained and validated yearly.

### Epidemiological investigation

**Figure 1:**
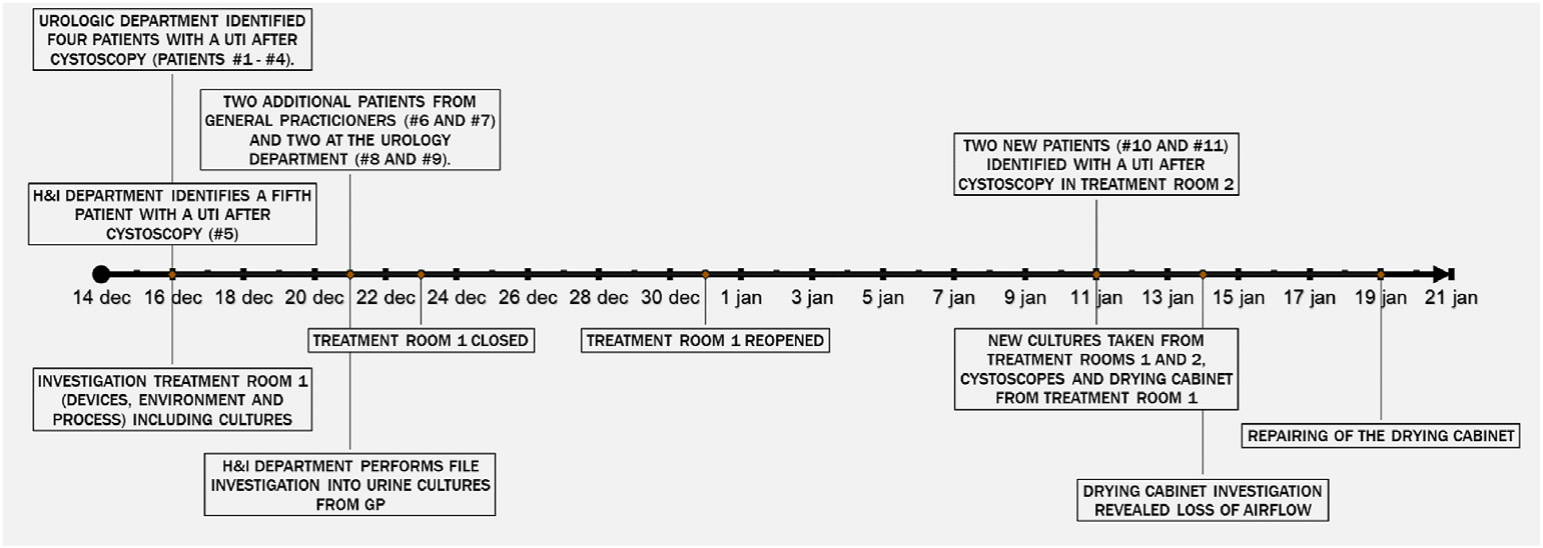
Timeline of identified patients with *Pseudomonas aeruginosa* urinary tract infection and events during the outbreak.

On December 16, 2021, the urology outpatient department reported a possible increased number of UTI in patients after cystoscopy (Figure 1). Four patients had undergone a cystoscopy in the period from December 3 to December 14, 2021 and subsequently developed symptoms consistent with a UTI. The urology outpatient department informed the department of hygiene and infection prevention (H&I department). This department identified another fifth patient who, after cystoscopy, had presented to the emergency department with a UTI. Electronic patient files of all five cases were reviewed by the H&I department to identify common factors as a starting point for an outbreak investigation.

### Bacterial cultures

Standard medical microbiological procedures based on the Clinical Microbiology Procedures Handbook of the American Society for Microbiology were used to culture bacteria from urine specimens, environmental samples, cystoscopes and drying cabinets [12].

### Amplified fragment length polymorphism

Comparative molecular typing of *P. aeruginosa* isolates was performed by means of amplified fragment length polymorphism (AFLP) using the icTyping assay (InBiome, Amsterdam, the Netherlands). With this assay, bacterial strains can be typed, based on restriction site distribution throughout their genome [13]. The digested and ligated bacterial DNA was amplified with fluorescently labeled, adaptor specific primers. PCR fragments were separated on an ABI 3130 Genetic Analyzer (Thermo Fisher Scientific, Bleiswijk, the Netherlands). AFLP patterns were analyzed with BioNumerics software package version 7.1 (Applied Maths, Sint-Martens-Latem, Belgium). Bands between 60 and 600 nucleotides were included in the analysis. Similarity coefficients were calculated with Pearson correlation and dendrograms were obtained by UPGMA clustering [14]. Isolates with a similarity of at least 97% were assigned a similar type. In total, 10 outbreak isolates were analyzed. *P. aeruginosa* strain ATCC27853 served as reference strain.

### Ethical statement

Informed consent was not obtained from patients involved in this outbreak. All patients were treated according to clinical judgement and infection control practices in order to treat them and control the outbreak according to local guidelines. Patients did not undergo randomization or intervention for the purpose of this report. Data has been analyzed and presented anonymously.

## Results

The investigation of the H&I department into the increased number of UTI in patients after cystoscopy revealed several factors in common among the five patients: all were male, all were infected with a *P. aeruginosa* bacterium with an identical antibiotic resistance pattern and all were treated in treatment room 1. Two patients were hospitalized for their UTI among which one case with *P. aeruginosa* bacteremia (Table 1).

**Table I:** Overview of 11 patients in order of subsequent identification related to the outbreak of *Pseudomonas aeruginosa* following cystoscopy

Patient	Sex	Age	Date scopeprocedure	Date urineculture	Urine culturebefore procedure	Admission	Bacteremia
1	m	67	3-12-2021	4-12-2021	No	No	No
2	m	77	8-12-2021	10-12-2021	No	Yes	Yes
3	m	71	10-12-2021	13-12-2021	14 days, neg	No	No
4	m	83	14-12-2021	16-12-2021	9 days, neg	No	No
*5*	m	77	4-11-2021	8-11-2021	No	Yes	No
*6*	m	75	1-11-2021	2-11-2021	No	No	No
*7*	m	72	23-11-2021	28-11-2021	No	No	No
*8*	m	65	5-11-2021	18-11-2021	No	No	No
9	m	64	15-12-2021	22-12-2021	22 days, neg	Yes	No
10	m	39	5-1-2022	6-1-2022	30 days, neg	Visit to emergencydepartment	No
11	m	73	10-1-2022	11-1-2022	8 days, neg	Yes	No

As treatment room 1 had been specifically identified as being associated with the risk of infection, the investigation initially focused on the inventory and equipment of that room: the devices used (cystoscopes), the environment in which they were used (treatment chair, sink and floor drains) and the actions of the users (processes). Three cystoscopes were taken out of circulation for a technical check. To investigate problems in the cleaning procedures, cultures of these cystoscopes were taken after cleaning and disinfection at the reprocessing unit for reusable medical devices before they were placed in the drying cabinet of treatment room 1. They were taken out of use until cultures were negative. Inspection of the treatment room by H&I workers, including environmental cultures and processes, resulted in recommendations for improvement of hygiene conditions.

Cultures from the sink drain of the treatment chair revealed growth of several Gram-negative rods, among which two *P. aeruginosa* isolates with different antibiotic resistance patterns compared to both each other and to patient isolates. The cultures from the three cystoscopes taken after disinfection at the reprocessing unit for reusable medical devices were negative upon which they were released for subsequent use.

On December 21, 2021, additional investigation of patient files identified another two male patients with *P. aeruginosa* cultured from urine specimens submitted by general practitioners following cystoscopy in Bernhoven. In addition, two other male patients with *P. aeruginosa* bacteriuria following cystoscopy were identified at the urology outpatient department. These two patients had undergone cystoscopy on November 5 and December 15, just before the urology outpatient department reported the possible outbreak. All nine patients had undergone cystoscopy at treatment room 1 and, therefore, on December 23, it was decided to close this room for patient procedures.

Subsequently, the sink of the treatment chair in treatment room 1 was disinfected at the hospital's sterilization department. A drain hose and coupler were replaced. Because of tears in the seat of the treatment chair, a new chair was ordered. Disinfection protocols were updated. Additional cultures were taken from the treatment chair and sink. They reported negative on December 31. Subsequently, treatment room 1 was re-opened for those occasions that a double program was planned. In case a single program was planned, only treatment room 2 was used.

On January 11, 2022, a *P. aeruginosa* isolate was reported in a urine culture from a tenth male patient with a UTI who had undergone cystoscopy at treatment room 2 on January 5. Likewise, an eleventh male patient was identified who had undergone cystoscopy on January 10 and already presented with *P. aeruginosa* UTI on January 11. Further investigation revealed that cystoscopes from the drying cabinet in treatment room 1 were used for cystoscopy in treatment room 2. Subsequently, environmental cultures were taken from both treatment rooms including the drying cabinet.

On January 14, a planned validation of the drying cabinet in treatment room 1 took place which revealed lack of airflow through the cystoscopes. The technical defect in the airflow had not been noticed before because of the absence of an alarm that detects loss of airflow. Cystoscopies were subsequently performed only with cystoscopes delivered directly from the reprocessing unit for reusable medical devices. Cultures were taken from four cystoscopes inside the drying cabinet of treatment room 1. All cultures from the cystoscopes inside the drying cabinet and draining of treatment room 1 were positive for *P. aeruginosa* with the same antibiotic resistance pattern as the patients with UTI, while cultures taken of surfaces inside the drying cabinet and from the treatment rooms were negative.

**Figure 2:**
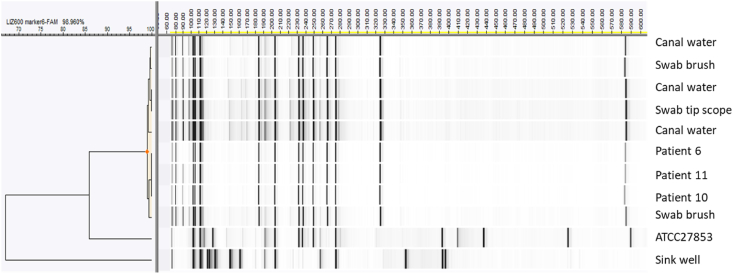
Dendrogram of cluster analysis of amplified fragment length polymorphism fingerprints of ten *Pseudomonas aeruginosa* isolates obtained during the outbreak. ATCC 27853 served as reference strain. Hierarchical clustering was performed using Pearson correlation and unweighted pair-grouping. The patterns of the ATCC strain and the *P. aeruginosa* isolate obtained from the sink drain of the treatment chair in treatment room 1 are clearly different compared to the other nine isolates tested. The three isolates obtained from the different patients and six isolates from cystoscopes showed a similarity of 99% which indicate that they belong to the same clone.

The genotypic relatedness among a selection of 10 isolates was determined by AFLP fingerprinting. *P. aeruginosa* strain ATCC 27853 was added to this selection as reference and unrelated strain. Clinical isolates from three patients as well as six strains from cystoscopes belonged to one single *P. aeruginosa* strain. The tenth *P. aeruginosa* strain from the sink drain of the treatment chair in treatment room 1 was not genotypically related to the other strains (Figure 2).

The drying cabinet in treatment room 1 was repaired on January 19.

On January 20, patients that had presented with a *P. aeruginosa* UTI following cystoscopy as well as all general practitioners referring to the hospital were informed of the outbreak. All drying cabinets throughout the hospital were inspected by the H&I department and technical service of the hospital.

On January 26, cystoscopes were placed back in the disinfected drying cabinet of treatment room 1. The next day, cultures were taken from the cystoscopes inside the cabinet which came back negative. On February 1, the drying cabinet of treatment room 1 was released for subsequent use. It is planned that the drying cabinet will be replaced for one with an alarming system. Until then, daily checks for the presence of airflow in the cabinet are performed.

## Discussion

*P. aeruginosa* is an important bacterial pathogen, particularly as a cause of nosocomial infections in hospitalized patients. Several outbreaks of *P. aeruginosa* caused by contaminated endoscopes have been reported [[5], [6], [7]]. Disinfecting scopes contaminated with *P. aeruginosa* can be challenging because of the ability of the bacteria to produce an exopolysaccharide biofilm that protects it from surfactant containing detergents [10].

In our hospital we noticed an outbreak of *P. aeruginosa* UTI in male patients after cystoscopy, caused by a malfunctioning airflow in the drying cabinet in treatment room 1. AFLP analysis identified one single clone of *P. aeruginosa.* How *P. aeruginosa* got in the drying cabinet system is not clear. Not all clinical strains of patients were available for further analysis, but we assume that the other eight patients were also infected with the same clone. The environment for growth in a drying cabinet is good for *P. aeruginosa* as conditions are moist and warm. The technical defect in the airflow in the drying cabinet had not been noticed before because of the absence of an alarm that detects loss of airflow. To our knowledge, this is the first report describing a malfunctioning drying cabinet as cause of an outbreak following cystoscopy.

Although contaminated endoscopes have been implicated in outbreaks of *P. aeruginosa*, only a few reports exists in which a cystoscope was involved [[8], [9], [10]]. Patients should be advised to observe and report the occurrence of urologic symptoms after urologic exploration. In case of concomitant infections caused by *P. aeruginosa*, the cystoscope should be suspected as a potential reservoir [8]. Molecular techniques are helpful in investigation of the epidemiology of an outbreak and in confirming clonality of outbreak strains [3].

## Conclusion and recommendations

Patients should always be informed of recognizing and reporting the possible urologic symptoms of a UTI after diagnostic or therapeutic procedures with urologic scopes. In case of concomitant infections caused by *P*. *aeruginosa*, the cystoscope and drying cabinet should be considered as a potential source. A drying cabinet with automatic detection of airflow loss (full process registration) is preferred. Daily manual checks are advised if the drying cabinet does not have any automatic detection. Storage of endoscopes in the drying cabinet is safe for up to 30 days before use. Recommendations for improvement of hygiene conditions in the treatment room must be recorded. Conduct regular audits and inspections of the general hygiene in the treatment room. Molecular techniques are helpful in investigation of the epidemiology of an outbreak.

## Credit author statement

Not relevant/applicable.

## Conflict of interest

The authors have no conflicts of interest to declare. All authors have seen and agree with the contents of the manuscript and there is no financial interest to report. We certify that the submission is original work and is not under review at any other institution. This research received no specific grant from any funding agency in the public, commercial, or not-for-profit sectors.
